# Lack of Association Between Peripheral Activity of Thyroid Hormones and Elevated TSH Levels in Childhood Obesity

**DOI:** 10.4274/Jcrpe.1251

**Published:** 2014-06-05

**Authors:** Denisa Lobotková, Daniela Staníková, Juraj Staník, Ol’ga Cervenová, Vladimír Bzdúch, L’ubica Tichá

**Affiliations:** 1 Comenius University Faculty of Medicine and Children’s University Hospital, Department of Pediatrics, Bratislava, Slovakia

**Keywords:** thyroid hormones, TSH, peripheral activity, children, obesity

## Abstract

**Ob­jec­ti­ve:** An elevated thyroid stimulating hormone (TSH) level is a frequent finding in obese children, but its association with peripheral hormone metabolism is not fully understood. We hypothesized that in obesity, the changes in thyroid hormone metabolism in peripheral tissues might lead to dysregulation in the thyroid axis. The purpose of this study was to investigate the association of TSH with thyroid hormones in a group of obese children as compared to normal-weight controls.

**Methods:** Serum TSH, free thyroxine (fT4) and free triiodothyronine (fT3) levels were measured in 101 obese children and in 40 controls. Serum reverse T3 (rT3) levels were also measured in a subgroup of 51 obese children and in 15 controls.

**Results:** Serum TSH level was significantly higher in obese children compared to controls (2.78 vs. 1.99 mIU/L, p<0.001), while no difference was found in fT4, fT3, rT3 levels and in fT3/rT3 ratio. In the obese group, fT3 level positively correlated with fT4 (r=0.217, p=0.033) and inversely with rT3 (r=-0.288, p=0.045). However, thyroid hormone levels and TSH levels were not correlated.

**Conclusion:** In obese children, normal fT4, fT3 and rT3 levels suggest an undisturbed peripheral hormone metabolism. These levels show no correlation with elevated TSH levels.

## INTRODUCTION

It is well known that thyroid hormones are largely involved in the regulation of metabolism, energy homeostasis and body weight. However, the role of thyroid hormones and especially of thyroid axis dysfunction in the pathogenesis of obesity is not clear. A large proportion of obese children have isolated elevation of serum thyroid stimulating hormone (TSH) level (1,2,3). The mechanism underlying this change is not yet fully understood. The altered thyroid function seems to be the consequence rather than the cause of the excess body fat, but might also contribute to difficulties with both weight reduction and maintaining weight loss (2). 

Several hypotheses have been advanced to explain the mechanisms leading to hyperthyrotropinemia, including variation in the activity of peripheral deiodinases leading to the possible changes of thyroid hormone action at the cellular level (4). For example, patients with anorexia nervosa, who represent the state opposite to obesity, often present with low free triiodothyronine (fT3) and elevated reverse T3 (rT3) levels, suggesting a protective mechanism for storing energy in the state of negative energy balance (5,6,7). The opposite situation has been described in obese patients by some authors, where over nutrition was associated with higher fT3 levels (1,6,8). However, studies documenting rT3 levels in obese children are scarce (9). In animal studies in diet-induced obese rats, increased rT3 instead of fT3 was reported, indicating presence of a mechanism that might impair the further increase in oxygen consumption (4,10). We hypothesized that a similar dysregulation might be present in obese children with elevated TSH levels. Therefore, the aim of this study was to compare the peripheral activity of thyroid hormones including rT3 levels and their association with TSH in groups of obese and normal-weight children.

## METHODS

**Patients**

101 obese (46 girls and 55 boys) and 40 healthy normal-weight (19 girls and 21 boys) children (control group) aged 4-18 years were recruited for the study. All the children were examined in the years 2011-2013 in the 1st Department of Paediatrics, Children’s University Hospital, Bratislava, Slovakia. Obesity was defined as a body mass index (BMI, weight in kg divided by the square of height in m) over 97th percentile according to the national BMI percentile charts (11). Patients with secondary causes of obesity, positive thyroid auto-antibodies and patients treated with thyroxine (T4) were excluded from the study. Informed consent for all procedures was obtained from the parents or legal guardians of the children before enrolment. The study protocol for rT3 (as it is not included in the standard laboratory tests) was approved by the Ethics Committee of the Children´s University Hospital in Bratislava. 

**Methods**

Anthropometric measurements were performed by trained nurses using standard scales with a height rod. BMI and BMI standard deviation score (SDS) were calculated in all subjects. Pubertal development was assessed by visual inspection using Tanner stages (12,13). After an overnight fasting, blood samples for serum hormonal analysis were collected from all participants [TSH, free T4 (fT4), fT3]. In addition, rT3 levels were measured in random subgroups of 51 obese and 15 control children. 

**Assays**

Serum TSH, fT4 and fT3 levels were measured by electrochemiluminescence immunoassay (ECLIA; Roche Diagnostics GmbH, Mannheim); assay sensitivity for these three parameters were 0.005 mIU/L, 0.30 pmol/L and 0.4 pmol/L, respectively. Isolated hyperthyrotropinemia was diagnosed when TSH level was over 4 mIU/L with normal fT3 and fT4 levels and negative antibody levels (anti-TPO-Ab ≤34.0 kIU/L and anti-TG-Ab ≤115.0 kIU/L) (2,3,14). Serum rT3 level was determined by commercial Human Reverse Triiodothyronine ELISA Kit (Cusabio) with assay sensitivity of 1.6 ng/mL; the equation rT3 ng/mL x 1.54=nmol/L was used for conversion from mass to SI units.

**Statistical Analysis**

Power and Sample Size: The power analysis was performed with the GraphPad StatMate 2.00 software for design with unequal sample sizes. For TSH, fT4 and fT3 between-group comparisons, the required minimum sample sizes were calculated for 80% power, α=0.05 type I error, β=0.2 type II error and effect size of d≥0.5 (medium size effect) as 76 patients in the obese group and 38 patients in the control group. For rT3 and fT3/rT3 between-group comparisons, the required minimum sample sizes were calculated for 80% power, α=0.05 type I error, β=0.2 type II error and effect size of d≥0.8 (large size effect) as 42 patients in the obese group and 14 patients in the control group. Additional 10%-30% patients were added into each group to protect the study from potential loss of data. 

The statistical analysis of the data was performed with the SPSS statistics 17.0 software. Normality of the data was assessed using Shapiro-Wilk test. The correlations between various variables were tested by the Spearman coefficient. Inter-group comparisons of quantitative variables were made by unpaired t-test for parameters with normal distribution (fT3, rT3) and Mann-Whitney test for not normally distributed data (TSH, fT4, BMI SDS). A p-value of less than 0.05 was considered statistically significant and 95% confidence intervals were calculated. 

## RESULTS

**Figure 1:**
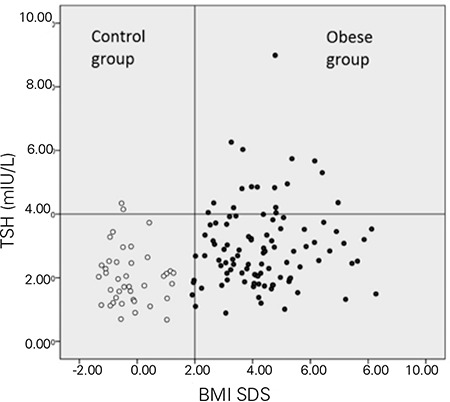
Thyroid stimulating hormone (TSH) serum levels in the obese and control groups

The median Z-score BMI (BMI SDS) was 4.21 (1.97) in the obese group and -0.465 (1.16) in the control group. The mean age of the children was 12.1±2.9 years in the obese group and 12.3±3.5 years in the control group. An elevated serum TSH level (≥4 mIU/L) was found in 17 (16.8%) obese children (10 girls, 7 boys) and in 2 (5%) children (boys) in the control group. None of the children had markedly elevated TSH levels (>10 mIU/L). Only 2 obese children and 1 child in the control group had slightly elevated serum fT3 levels. Serum fT4 level was within the reference range in all the children in both groups. Data pertaining to thyroid function in obese children and the control group are summarized in Table 1. The TSH level was significantly higher in the obese children (2.78 vs. 1.99 mIU/L, p<0.001) as seen in Figure 1.

A positive correlation was found between TSH and BMI SDS (r=0.323, p<0.001), when obese children and controls were taken together. Mean fT4, fT3, rT3 levels and fT3/rT3 ratio were slightly higher in the obese children; however, this difference was not significant (p=0.610 for fT4; p=0.228 for fT3, p=0.100 for rT3 and p=0.792 for fT3/rT3). Obese children with elevated serum TSH levels (≥4 mIU/L) did not differ in fT4, fT3, rT3 and fT3/rT3 ratio compared to the rest of obese children with normal TSH levels (Table 2).

In obese children, TSH values did not correlate with fT4 and fT3, neither in children with normal nor in children with elevated TSH levels. Serum fT3 was correlated positively with fT4 (r=0.217, p=0.033) and inversely with rT3 (r=-0.288, p=0.045) in the obese children.

## DISCUSSION

Reported figures on prevalence of hyperthyrotropinemia (serum TSH level above 4 mIU/L) in obese children vary from 22.2% to less than 2% (2,15,16). In the present study, elevated serum TSH levels (≥4 mIU/L) were found in 16.8% of obese children and adolescents with negative thyroid antibodies. Positive thyroid auto-antibodies have been reported by other authors to be present in up to 20% of obese children with high serum TSH levels (1,3,14,17,18). Serum TSH levels of the obese children in our series were also significantly higher compared to those of the controls and correlated positively with BMI SDS, a finding which is in agreement with previously published data (1,3,8).

Several possible explanations for the elevated TSH levels in obese subjects have been discussed in the literature, with leptin being the most probable link between thyroid function and weight status. Leptin is known to affect the activity of the hypothalamic-pituitary-thyroid axis both directly and indirectly. Serum leptin levels are increased in the state of positive energy balance. Leptin also acts as an important modulator of central and peripheral iodothyronine deiodinases, which are involved in the control of thyroid hormone action and biological availability of T3 at the cellular level (4,6,15,19,20).

Alterations in iodothyroinine deiodinases have been suggested to play a role in the disruption of the normal hypothalamic-pituitary-thyroid axis in obesity (4,21). Deiodinases are responsible for deiodination of T4 to its active metabolite T3 (D1, D2) or to its inactive product rT3 (D3). In normal-weight humans, monodeiodination of T4 produces approximately equal amounts of T3 and rT3. In obesity, production of T3 has been reported to be normal or increased (21,22), while data on rT3 are scarce and inconsistent (4,9,23,24). T3 increases energy expenditure in the organism and as a consequence, the availability of accumulated energy for conversion into fat is diminished. Since rapid weight loss is associated with a decrease in both TSH and T3 levels, the resulting decrease in resting energy expenditure (REE) may contribute towards the difficulties maintaining weight loss (6,22). This theory is supported by the results of Wolters et al (25) which indicate that the decrease in TSH and fT3 concentrations during lifestyle intervention is associated with weight regain after the intervention. 

Conversely, in rodents with a high-fat diet-induced obesity, instead of increased serum T3, an elevated serum rT3 was reported, which is a mechanism that might impair the further increase in oxygen consumption (26). Increased serum rT3 could behave as a physiological inhibitor of pituitary D2 activity leading to local hypothyroidism which in turn leads to increased TSH secretion (4,10). As data on production of rT3 in obese children are missing, we decided to measure rT3 levels in subgroups of obese children and normal-weight controls. Our results showed that mean fT3 and also rT3 levels were slightly higher in the obese group, but this difference was not significant, while there was a negative correlation between fT3 and rT3. These results represent a normal state of peripheral thyroid hormones conversion, therefore are not in agreement with the above-mentioned animal studies. However, we cannot rule out possible differences in animal and human metabolism and also the interference of the different nutritional diet composition on thyroid function during positive energy balance.

Iodine deficiency has been suggested as one of the possible causes of increased TSH level in obese children, but the results reported by Stichel et al (3), which show that urinary iodine excretion is normal in most obese children, do not support this theory. We did not measure urinary iodine in our study, but according to official reports, iodine intake of the Slovak Republic population is equal to or slightly above the recommended dietary allowance (27) and urinary iodine values in the Slovak population including children are within the optimal range (>100-200 µg/L) (28,29). Therefore, we do not suggest iodine deficiency to be a factor responsible for the increased TSH levels in the obese children in our study. 

As there is a dilemma about the need for pharmacologic treatment for elevated TSH levels by L-T4 to prevent overt hypothyroidism in obese children, a question arises on whether the elevated TSH level represents a hypothyroid state in the organism. In previous studies, total and also fT3 levels of obese children with elevated TSH levels were reported to be significantly higher compared to the control group (obese children with normal TSH levels), while T4 levels did not differ significantly (1,3,30). A positive correlation between TSH and fT3, but not between TSH and fT4 and a negative correlation between fT4 and fT3 was found in a study on underweight, normal-weight and obese female adolescents (6). Contrary to these findings, in the present study, we found no difference in fT3 levels neither between obese children and the control group, nor among the subgroups of obese children with elevated and normal TSH levels. According to our results, the peripheral conversion of fT4 to fT3/rT3 seems to be undisturbed in obesity and is not correlated to elevated TSH. These results are in agreement with the theory that elevated TSH levels observed in obese children represents possible hormone resistance and disturbed negative feedback and not hypothyroidism per se (3,21). Pharmacologic treatment with T4 thus seems unnecessary in obese children (2,31). This opinion is supported by the work by Eliakim et al (14), who demonstrated that elevated TSH levels normalize after weight loss. 

However, in the presence of obesity, “normal” levels of thyroid hormones may be simply not “enough” to deal with the unhealthy positive energy balance and the “exact” peripheral activity is not really known. Thus, future studies involving changes in metabolism such as REE in obesity and subsequent weight loss and the association of these changes with thyroid hormones as well as the activity of central and peripheral deiodinases are needed. 

In conclusion, a moderate elevation of serum TSH level is frequently found in obese children, but this finding is not linked to changes in the peripheral activity of thyroid hormones. The peripheral activity seems to be undisturbed as shown by normal concentrations of thyroid hormones and normal conversion of fT4 to fT3/rT3 in obese children. 

**Acknowledgements**

We would like to thank Kyselova T, Dr. and Semberová J, MD, PhD. for rT3 determination.

**Funding**

This work was partially supported by the Grant of Comenius University (grants for PhD. students and young scientific researchers of CU) GUK No. 355/2011.

## Figures and Tables

**Table 1:**
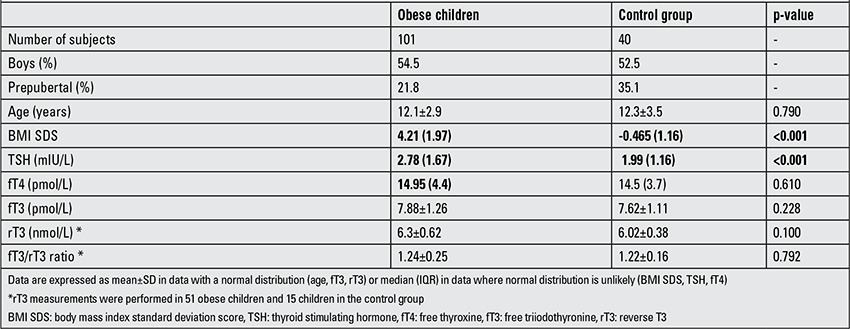
Basic characteristics and thyroid hormone levels in obese children and in the control group

**Table 2:**
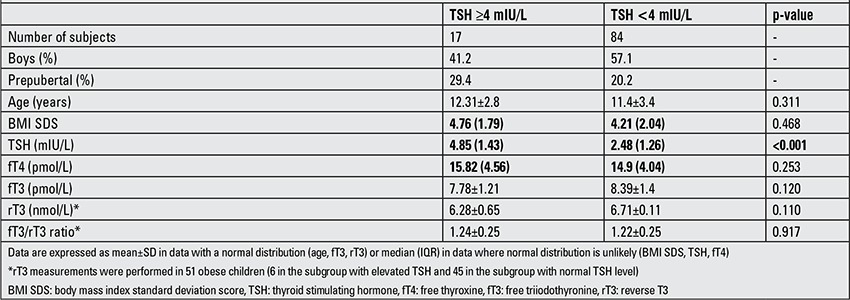
Basic characteristics and thyroid hormone levels in subgroups of obese children with elevated (≥4 mIU/L) and normal (<4 mIU/L) serum TSH levels
